# Proper direction of male genitalia is prerequisite for copulation in *Drosophila*, implying cooperative evolution between genitalia rotation and mating behavior

**DOI:** 10.1038/s41598-018-36301-7

**Published:** 2019-01-18

**Authors:** Momoko Inatomi, Dongsun Shin, Yi-Ting Lai, Kenji Matsuno

**Affiliations:** Osaka University, Graduate School of Science, Department of Biological Sciences, Osaka, 560-0032 Japan

## Abstract

Animal morphology and behavior often appear to evolve cooperatively. However, it is difficult to assess how strictly these two traits depend on each other. The genitalia morphologies and courtship behaviors in insects, which vary widely, may be a good model for addressing this issue. In Diptera, phylogenetic analyses of mating positions suggested that the male-above position evolved from an end-to-end one. However, with this change in mating position, the dorsoventral direction of the male genitalia became upside down with respect to that of the female genitalia. It was proposed that to compensate for this incompatibility, the male genitalia rotated an additional 180° during evolution, implying evolutionary cooperativity between the mating position and genitalia direction. According to this scenario, the proper direction of male genitalia is critical for successful mating. Here, we tested this hypothesis using a *Drosophila Myosin31DF* (*Myo31DF*) mutant, in which the rotation of the male genitalia terminates prematurely, resulting in various deviations in genitalia direction. We found that the proper dorsoventral direction of the male genitalia was a prerequisite for successful copulation, but it did not affect the other courtship behaviors. Therefore, our results suggested that the male genitalia rotation and mating position evolved cooperatively in *Drosophila*.

## Introduction

The evolution of animal behaviors often appears to be accompanied by morphological changes, suggesting that the behavior and morphology evolved cooperatively^1–6^. One well-known example of such evolutionarily cooperativity is the relationship between the beak shape of Darwin’s finches and their various eating habits^1,3^. These birds show variations in food sources, including the nectar and pollen of flowers, fruits, seeds, insects, snails, and even the blood of seabirds^1,3^, and their beak shapes vary depending on their eating habits^1,3^. Furthermore, the genes responsible for the evolutionary diversification of the beak morphology have been identified^1,2^. Therefore, morphological changes that appear to show cooperative evolution with behaviors can be attributed to genomic modification. If the beak shapes, but not the behaviors associated with eating habits, changed, such changes would not make sense and *vice versa*. This logic leads to the idea that morphology and behavior need to evolve cooperatively. However, it has been difficult to assess to what extent organ morphology is critical for and couples with a particular animal behavior, mostly because the experimental modification of organ morphology is difficult to achieve without generating other side effects.

**Figure 1:**
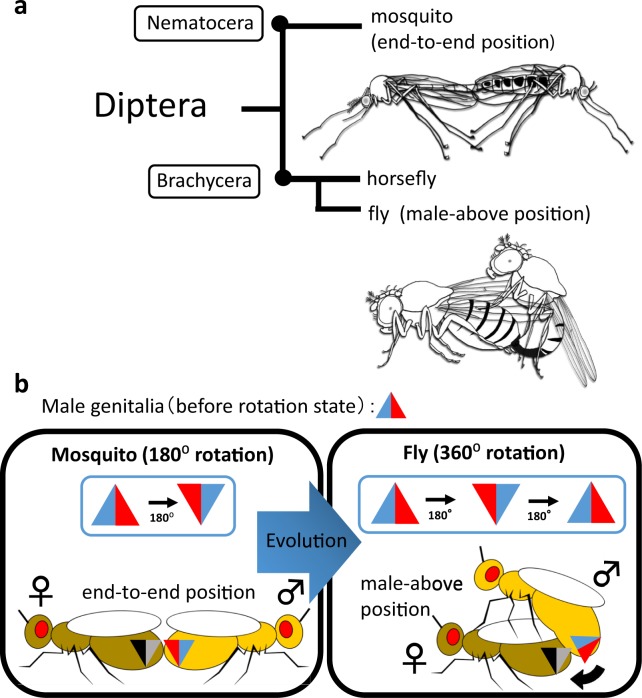
Evolutionary relationship between mating position and male genitalia rotation in dipterans. (**a**) Phylogenetic tree showing the evolution of mating positions in Diptera. Illustrations show the mating of mosquitos (upper, end-to-end position) and flies (bottom, male-above position). (**b**) Hypothesis for the cooperative evolution between mating position and male genitalia rotation. Red-blue triangles show male genitalia, and black-grey triangles represent female genitalia. If the male genitalia rotate 180°, their dorsoventral direction becomes upside down. Under this condition, the dorsoventral directions of the male and female genitalia are in accordance in the end-to-end position of mosquitoes (left). However, in the male-above position, this relative dorsoventral direction of the male genitalia with respect to those of the female would be upside down. It was previously proposed that this inconsistency in the dorsoventral direction of the female and male genitalia could be overcome by an additional 180° rotation of the male genitalia in flies (right). This hypothesis implies that cooperative evolution occurs between the mating position and the rotation of male genitalia.

Insect species exhibit a great variety of mating behaviors^4^. Given that insects also have large variations in copulatory organ morphologies, insects could be particularly useful for studying the cooperative evolution between mating behavior and genitalia morphology. For example, insects have at least five typical mating positions: end-to-end, male-above, female-above, belly-to-belly, and false-male above^4^. In the end-to-end position mating, a male and a female copulate end-to-end in opposing directions through copulatory organs located at their posterior ends (Fig. 1a, upper and 1b, left). In the male-above position mating, a male mounts a female, and they copulate via their copulatory organs at the posterior end (Fig. 1a, lower and 1b, right).

The order Diptera encompasses species with one pair of wings and halters, including flies and mosquitos (Fig. 1a)^6^. Primitive dipteran species like mosquitos tend to mate in the end-to-end position, while the male-above position is common in higher dipterans, such as flies, suggesting that the end-to-end position is ancestral to the male-above position (Fig. 1a)^4–6^. Upon the presumptive evolution from the end-to-end to the male-above position, the dorsoventral direction of the male genitalia became reversed with respect to that of the female genitalia (Fig. 1b). Therefore, the genitalia direction needed to be modified so that the male and female copulatory organs would physically couple. It was proposed that one way to solve the incompatibility in the dorsoventral direction of the genitalia was to rotate the male copulatory organ (Fig. 1b)^4–6^. Interestingly, in many dipteran species, the male genitalia rotate to a certain angle during the pupal or adult stage, temporarily or permanently^4–6^. In species that show temporary rotation, the genitalia usually have some torsional flexibility^4,7^, while the rotation angle is fixed in species in which the male genitalia rotate permanently^7–9^.

In Diptera, the rotation angle of the genitalia is well correlated with the mating positions. A 180° rotation is found in species that mate in the end-to-end position or its derivatives. For example, the common house mosquito *Culex pipiens* mates end-to-end^10^ (Fig. 1b, left), and the yellow fever mosquito, *Aedes aegypti*, uses a belly-to-belly position, which is a variant of the end-to-end position and keeps the same dorsoventral direction of the male genitalia^4,5,11^. On the other hand, in the fruit fly *Drosophila melanogaster*, the male genitalia rotate 360° clockwise as viewed from the posterior end, and the male-above position is used (Fig. 1b, right). Thus, the additional 180° rotation of the male genitalia in *Drosophila* (*D*.) *melanogaster* appears to compensate for the discordance of the male genitalia direction in flies in the evolution from the end-to-end to the male-above position (Fig. 1b). These observations led to the proposal that incompatibility in the dorsoventral direction of the genitalia, which is associated with the evolution of the mating position, may be overcome by rotating of the male genitalia^6^. According to this scenario, the evolution of morphology (genitalia rotation) and behavior (mating position) could have occurred cooperatively (Fig. 1b).

Thus, assuming that the evolution of the genitalia rotation and mating positions occurred cooperatively, it is important to know how strictly these two traits depend on each other. If these two traits are very tightly interdependent, it is possible that an intermediate step occurred between the cooperative evolution of the genitalia rotation and mating position. However, although the angle of the genitalia rotation is precisely fixed in flies and mosquitoes, it is possible that mating behavior is flexible enough to overcome discordance in the dorsoventral direction of the genitalia. Here we addressed these issues using *D. melanogaster* as an experimental model.

The mechanisms underlying the male genital rotation have been studied extensively in *D. melanogaster*^7,9^. In particular, the directional rotation of the male genitalia is an excellent system for studying the mechanisms of left-right asymmetric development^7,9^. In the pupal stage, the 360° clockwise rotation is achieved by a combination of 180° rotations in two adjacent abdominal segments, abdominal segment 8 anterior and posterior^7,9^. During studies of left-right asymmetric development in this species, *Myosin31DF* (*Myo31DF*) mutants were identified, in which the left-right asymmetric development is reversed in various organs, including the male genitalia rotation^7,12–16^. *Myo31DF* encodes a *Drosophila* ortholog of Myosin ID^12,13,15,16^. The null mutation of *Myo31DF* is homozygous viable and fertile^14,15^. In most adult males homozygous for null or loss-of-function alleles of *Myo31DF*, the male genitalia rotate 360° counterclockwise^7,12–14^. Therefore, the roles of *Myo31DF* are specific to the left-right asymmetric development to some extent, although recent studies revealed that *Myo31DF* also plays essential roles in other cellular events^17,18^. Importantly, in addition to the inversion of the rotation direction, the rotation of the male genitalia is sometimes prematurely terminated in *Myo31DF* mutant flies^14^. Therefore, we can obtain male adult flies with genitalia that have rotated to various angles, from 0° (no rotation) to 360° (full rotation; circumversion), in the same genetic background.

In this study, using these *Myo31DF* mutant flies, we analyzed whether the normal dorsoventral direction of the male genitalia is a prerequisite for successful mating in the male-above position in *D. melanogaster*. We found that successful copulation required a certain degree of accuracy in the dorsoventral direction of the male genitalia. Our results suggest that the male–above position of this species evolved cooperatively with the additional 180° rotation of the male genitalia from the ancestral end-to-end position, as previously proposed based on the phylogenic relationship between mating behavior and male genitalia rotation^6,7^.

## Results

### *Myo31DF* mutant male flies have genitalia with angle deviations

**Figure 2:**
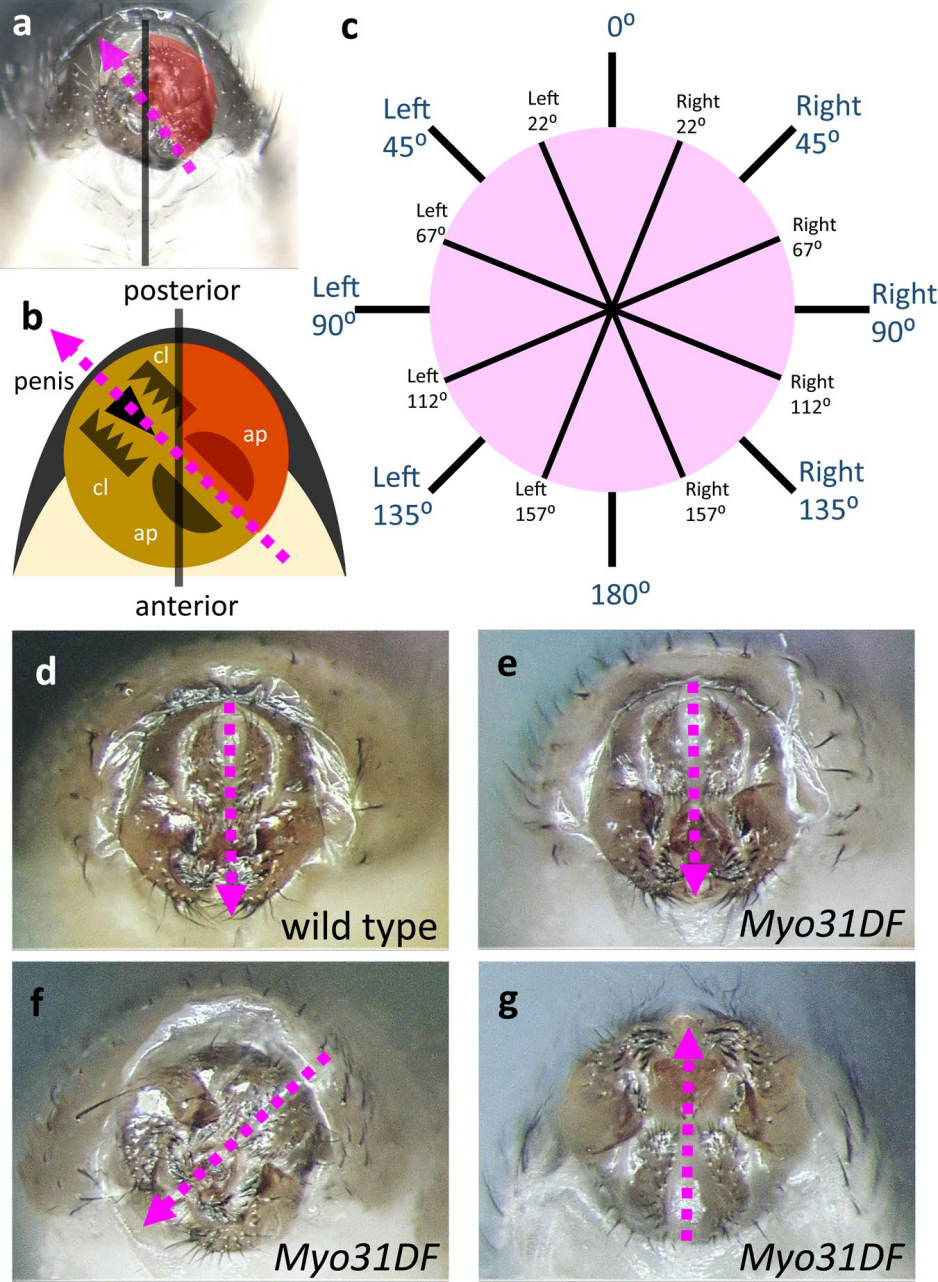
Classification of male genitalia with angle deviation. (**a** and **b**) Definition of the angle deviation in male genitalia. We measured the angle (shown in red) between the midline of the abdomen (solid black line) and the line connecting the anus and penis (broken magenta line). A photograph of male genitalia with the angle deviation (**a**) and its schematic representation (**b**) are shown. cl, clasper; ap, anal plate. (**c**) Eight classes of angle deviation in male genitalia: 0° (Left 22°-Right 22°), Right 45° (Right 22°-Right 67°), Right 90° (Right 67°-Right 112°), Right 135° (Right 112°-Right 157°), 180° (Right 157°- Left 157°), Left 135° (Left 157°-Left 112°), Left 90° (Left 112°-Left 67°), Left 45° (Left 67°-Left 22°). (**d**–**g**) Male genitalia of wild-type (**d**) and of *Myo31DF*^*K2*^ mutant (*Myo31DF*) flies with 0° (**e**), Right 45° (**f**), and 180° (**g**) angle deviation. The head of the magenta arrow shows the penis, and the tail indicates the anus in (**d**)–(**g**).

In *Myo31DF* homozygotes, the rotation of the male genitalia in some individuals stopped at various angles, resulting in male genitalia with “angle deviation” (Fig. 2). In this study, we obtained male flies with genitalia showing angle deviation in homozygotes of *Myo31DF*^*K2*^, a null mutant allele of *Myo31DF* that is homozygous viable and fertile^13,14^.

To evaluate how much the angle deviation of the male genitalia influenced reproduction efficiency, we classified the male genitalia with various degrees of angle deviation into eight categories (Fig. 2a–c). As schematically shown in Fig. 2b, we first defined the dorsoventral line of the male genitalia, which passed through the anus and penis (middle of claspers) (broken arrow in Fig. 2a,b). We then measured the angle formed by the midline of the abdomen (solid line in Fig. 2a,b) and the dorsoventral line of the male genitalia (broken arrow in Fig. 2a,b). Based on these angles, we defined 8 classes of angle deviation (classes: 0°, Right 45°, Right 90°, Right 135°, 180°, Left 135°, Left 90°, Left 45°) (Fig. 2c). Figure 2d–g shows typical examples of male genitalia with normal and deviated angles.

### Proper dorsoventral direction of the male genitalia is a prerequisite for successful reproduction

We next analyzed the success rate of reproduction involving the male flies with genitalia belonging to each of the 8 classes of angle deviation obtained from *Myo31DF*^*K2*^. A single male belonging to each class was mated with three virgin females. Following the removal of each male, the females were kept in the medium to allow egg-laying for another five days. The females were then discarded, and the medium was incubated for another five days. If offspring were found in the medium, the mating was judged as successful.

**Figure 3:**
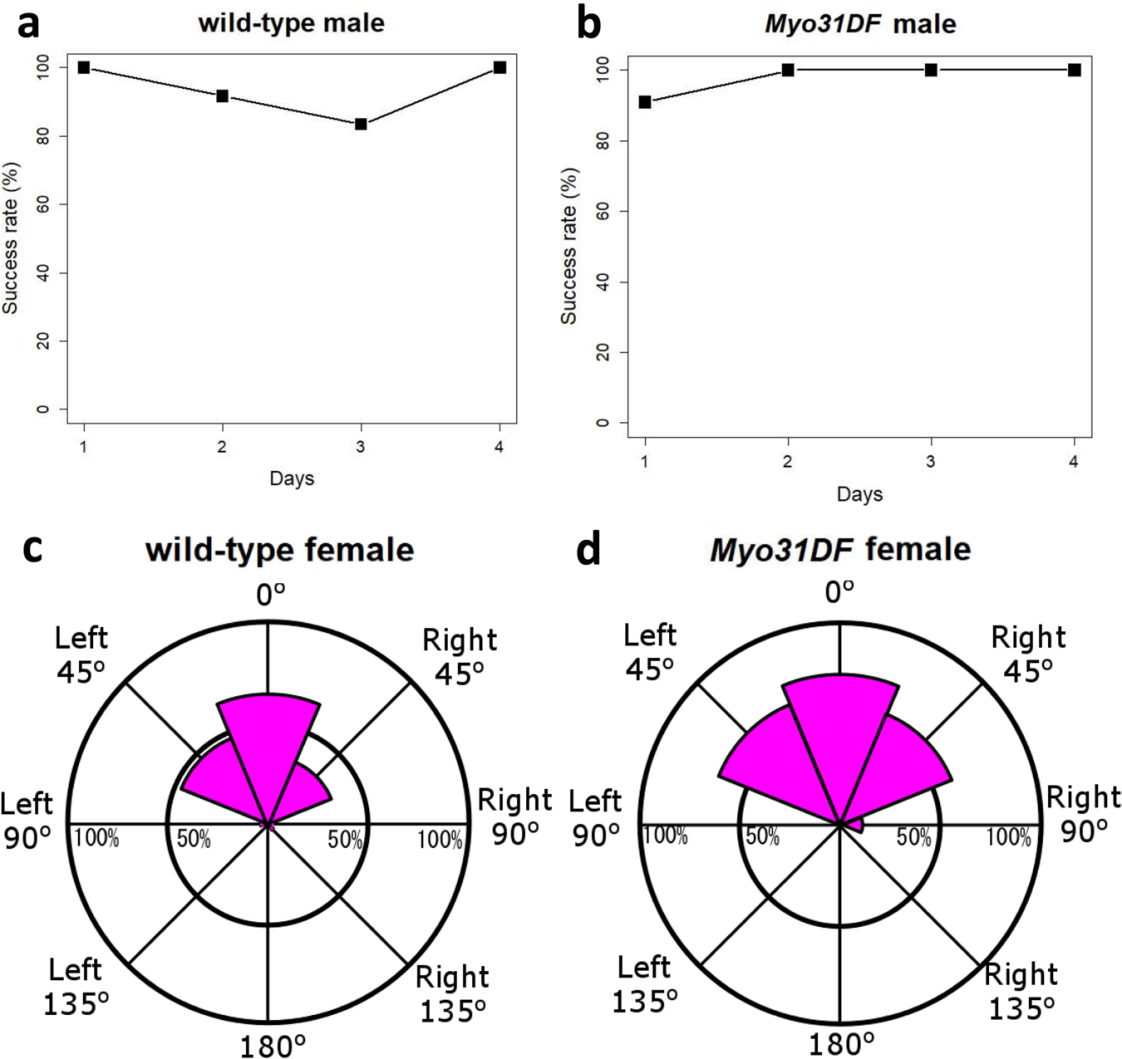
Males with genitalia exhibiting less angle deviation had an advantage in reproduction. (**a**,**b**) Reproduction success rates (%) after mating for 1, 2, 3, and 4 days. A single wild-type male (**a**) or *Myo31DF*^*K*2^ male with genitalia in the normal dorsoventral direction (**b**) was mated with three wild-type virgin females. (**c** and **d**) Reproduction success rates (%) for males with 8 classes of genitalia angle deviation, after mating for 4 days. Virgin females used for the mating were wild type (c) or *Myo31DF*^*K*2^ homozygotes (**d**).

First, we validated that our experimental conditions were sufficient for reproduction between wild-type females and males with genitalia of the normal direction (Fig. 3a,b). Single wild-type males or *Myo31DF*^*K2*^ homozygous males with genitalia in the normal direction (the genitalia rotated counterclockwise, but their final dorsoventral direction was normal) were mated with wild-type females for 1, 2, 3, and 4 days, followed by the recovery of offspring as described above. In all cases, the success rate of reproduction was more than 80% (Fig. 3a,b, Supplementary Table S1). We decided to use a 4-day mating period, because the success rate of reproduction was 100% in these two types of males (Fig. 3a,b and Supplementary Table S1).

Using the above experimental conditions, we next examined the reproduction rates using males with genitalia belonging to each of the eight classes of angle deviation (Fig. 3c,d). As shown in Fig. 3c, matings between wild-type females and males with genitalia of the 0°, Right 45°, Right 90°, Right 135°, Left 90°, and Left 45° angle deviations led to offspring at rates of 66.2, 33.9, 1.96, 3.18, 3.03, and 46.2%, respectively, whereas the males with genitalia of the 180° and Left 135° angle deviation did not have any offspring (Fig. 3c and Supplementary Table S2). Matings between *Myo31DF*^*K2*^ homozygous females and males with genitalia of the 0°, Right 45°, Right 90°, and Left 45° angle deviations led to offspring at rates of 75.0, 60.0, 9.80, and 65.0%, respectively, whereas the males with genitalia of the Right 135°, 180°, Left 135°, and Left 90° angle deviation did not have any offspring (Fig. 3d and Supplementary Table S2). Therefore, mating success strongly favored males with no or small angles of deviation (classes of the 0°, Left 45°, and Right 45° angle deviations). The same tendencies were observed with wild-type and *Myo31DF*^*K2*^ homozygous females, indicating that the strong reproductive superiority of the normal dorsoventral genitalia direction did not depend on a specific genetic background. Although males with genitalia in the normal direction yielded offspring at 100% frequency under these experimental conditions (Fig. 3a,b), males with the class 0° angle deviation had offspring only at rates of 66.2 and 75.0% with wild-type and *Myo31DF*^*K2*^ homozygote females, respectively (Fig. 3c,d and Supplementary Table S2). These results suggested that even a small angle deviation that was less than 22° decreased the reproduction success rate with wild-type and *Myo31DF*^*K2*^ homozygous females, with statistical significance in the case of wild-type females (Fisher’s exact test, *P* = 0.02879 for wild type and *P* = 0.1026 for the *Myo31DF*^*K2*^ homozygote).

Although males that had genitalia with the normal or almost normal direction had a great advantage for successful reproduction, we also found that males with an angle deviation of 45° or more still had offspring. In particular, males with genitalia with an angle deviation more than 67° (see Fig. 2c) still had offspring, although their success rate was very low (Supplementary Table S2). These results suggested that the males may have some ability to adjust the angle of their genitalia for copulation, although we cannot exclude the possibility that females contribute to this adjustment.

We also noted that males with the Left 45° versus Right 45° angle deviations did not show a marked difference in their reproduction success rate (Fig. 3c,d). In *Myo31DF*^*K2*^ homozygous males, the genitalia rotate counterclockwise^12,14^. Therefore, the genitalia only slightly rotated in males with the Left 45° angle deviation, but they rotated until slightly short of completion in males with the Right 45° angle deviation; that is, the Right 45° and Left 45° angle deviation was achieved by a 315° and 45° counterclockwise rotation, respectively. However, despite the 270° difference in rotation angle, the males with genitalia belonging to these two classes of angle deviation showed similar reproduction success rates with wild-type or *Myo31DF*^*K2*^ homozygous females (Fisher’s exact test, *P* = 0.3349 for wild type, *P* = 0.7921 for *Myo31DF*^*K2*^) (Fig. 3c,d). Thus, we speculated that the final direction of the genitalia, but not the genitalia rotation itself, had a significant physiological role in the success of reproduction.

### Angle deviation of the male genitalia reduces the success rate of copulation

**Figure 4:**
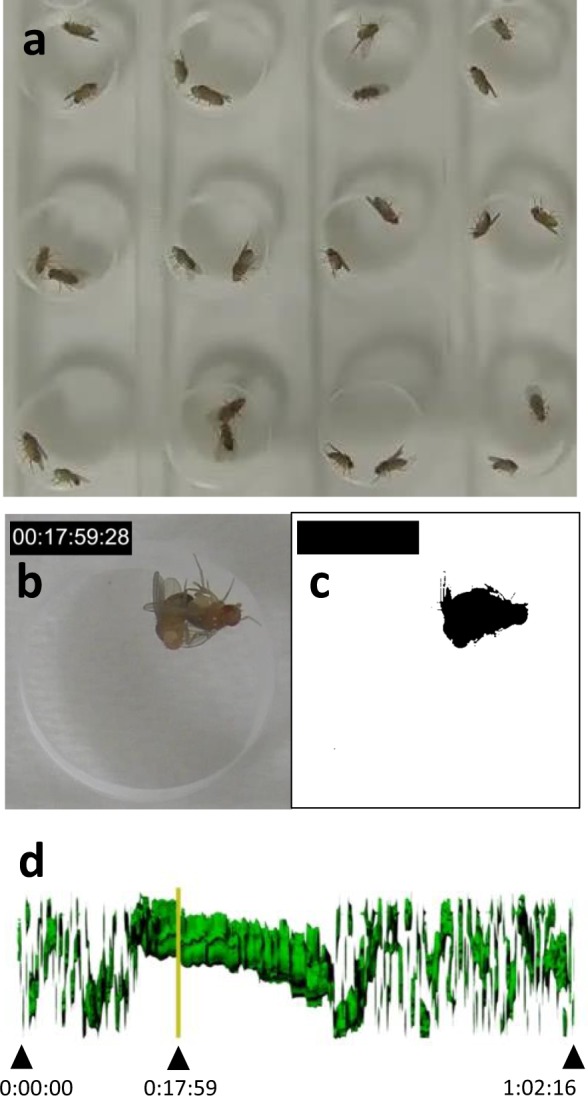
Analysis of courtship behavior using a novel image-processing program. (**a**) A snapshot of the video data of mating behavior between a virgin wild-type female and a male with genitalia with angle deviation or a control male with genitalia in the normal dorsoventral direction. (**b** and **c**) Snapshot of the original video (**b**) and its corresponding binarized picture (**c**) from the video recording of mating behavior in a single chamber. (**d**) Processed image in which the binarized images were automatically arranged in 3D space along a time line. A moving bar (yellow line) that was synchronized with the 2D image in b, c was inserted into the 3D image. Black triangles show time points.

The mating behavior of *Drosophila* consists of multiple steps, including orientation, tapping, following, singing (wing vibration), licking, and attempted copulation (mounting trial), before copulation^19,20^. Thus, to understand how the proper dorsoventral direction of the male genitalia affects reproduction success, we next investigated which step of the mating behavior was obstructed by the angle deviation of the male genitalia. Among them, copulation appeared the most likely to be sensitive to an abnormality in the dorsoventral direction of the genitalia. Thus, we first examined the success rate of copulation between wild-type females and males with genitalia with angle deviations classified as 0°, Right 45°, and 180°, obtained from *Myo31DF*^*K2*^ stock, and a control (*w*^1118^ otherwise wild type). A single virgin female was placed in a standard mating chamber, followed by the addition of a single male with genitalia at a normal or deviated angle (Fig. 4a). To analyze the mating behavior quantitatively, we video-recorded them for one hour after the addition of the male. To facilitate the video analysis, we developed a program to quickly identify the period of copulation. In this program, the image of flies (Fig. 4b) was binarized (Fig. 4c), and the shape of the flies was automatically selected and arranged in 3D space, where the binarized images were stacked along a time line (Fig. 4d and Supplementary videos 1–3).

**Figure 5:**
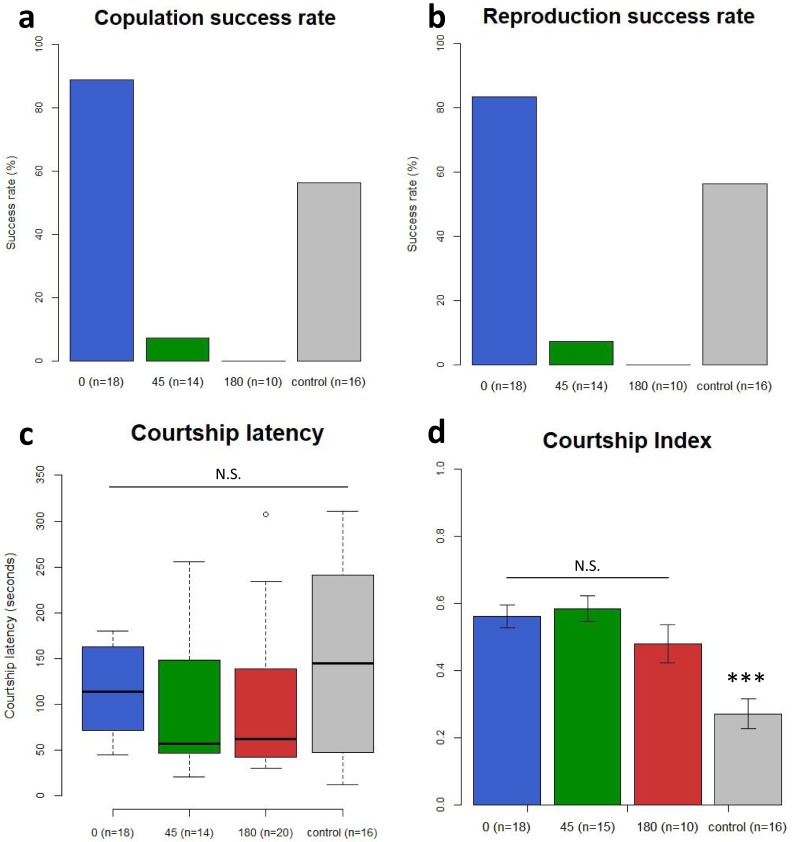
Males with genitalia exhibiting less angle deviation had more successful copulation. (**a**) Copulation success rates (%) of males with genitalia with angle deviation classified as 0° (0), Right 45° (45), and 180° (180) and of control males (control). (**b**) Reproduction success rates (%) of males with genitalia with angle deviation classified as 0° (0), Right 45° (45), and 180° (180) and of control males (control). (**c**) Courtship latency (seconds) of males with genitalia with angle deviation classified as 0° (0), Right 45° (45), and 180° (180) and of control males (control), shown as boxplots. In each boxplot, the middle band is the median (50^th^ percentile), and the length of the box shows the 1^st^ and 3^rd^ quartile (25^th^ and 75^th^ percentile). (**d**) CI of males with genitalia with angle deviation classified as 0° (0), Right 45° (45), and 180° (180) and of control males (control). The mean and standard error (error bars) are shown. In a-d, all the females were wild type, and the number of examined males is shown in parentheses as n. ***P-value < 0.01. N.S., not significant statistically.

In this analysis, a mating was defined as successful copulation if the male stayed on the back of a female for more than one minute during the one-hour experimental period. In the images processed by our program, the shapes of female and male flies partially overlap each other during copulation (Fig. 4d). We can then find the pictures from the video that correspond to “slices” in the 3D space; the yellow line in Fig. 4d corresponds to the pictures shown in Fig. 4b and c. We confirmed that using this program, we could accurately detect copulation events and determine the period of copulation using the processed image data from the video. If the male did not start wing vibration within 10 minutes, we did not include these data in the subsequent analysis, according to previous reports^21,22^. In our experiments, however, most of the males started the wing vibration (Supplementary Fig. S1 and Supplementary Table S3). Using the video data and image-analysis program, we found that males with the 0° angle deviation successfully copulated at 88.9% (Fig. 5a and Supplementary Table S4). We also found that the copulation rate of the control males, otherwise wild-type males whose genetic background was normalized to *Myo31DF*^*K2*^ by backcrosses, was 56.3% (Fig. 5a and Supplementary Table S4). However, the rate of successful copulation was severely reduced if the male had genitalia classified as Right 45° (7.14%) and 180° (0%) angle deviation (Fig. 5a and Supplementary Table S4). Therefore, the normal dorsoventral direction of the male genitalia was a great advantage for successful copulation. To confirm that our definition of successful copulation (a male mounting a female for more than one minute) was correlated with fecundity, we recovered each female from the chamber and examined whether she left offspring in the standard medium (Fig. 5b and Supplementary Table S4). In these experiments, all of the pairs of single females and males were found to copulate only once during one-hour period (Supplementary Fig. S2 and Supplementary videos 1 and 2). We found that males with genitalia classified as the 0° and Right 45° angle deviation and the control male had offspring at 83.3, 7.14, and 56.3%, respectively (Fig. 5b and Supplementary Table S4). These results showed a very similar tendency to the results of the copulation success rates (compare Fig. 5b with Fig. 5a). We also found that males with genitalia classified as 0° and Right 45° angle deviation and the control males that successfully copulated in the courtship assay had offspring at 93.8, 100, and 100% frequency, respectively (Supplementary Table S5). On the other hand, males with genitalia classified as 0°, Right 45°, and 180° angle deviation and the control males that failed to copulate in the courtship assay, did not have any offspring in any case (Supplementary Table S6). These results demonstrated that the copulation success rate showed a very good correlation with fecundity. Therefore, we concluded that the superiority of the males that had genitalia in the normal dorsoventral direction in having offspring (Fig. 3), was mostly attributed to a high success rate of copulation.

### Deviation in the genitalia direction does not affect courtship behaviors

Although we ascertained that the reduction in successful copulation was the major cause of the decrease in fecundity associated with the angle deviation of the male genitalia, it was still possible that other courtship behaviors were also influenced by the angle deviation of the genitalia. Using the video images, we measured the start time of courtship (first wing vibration) for the first 10 minutes after adding a single male that had genitalia with 0°, Right 45°, or 180° angle deviation or a single control male (backcrossed *w*^1118^) to the standard mating chamber containing a virgin female (Fig. 5c). This measurement was designated as the “courtship latency,” as described previously^21–23^. We found that the majority of these males initiated wing vibration within the first 10 minutes (Supplementary Fig. S1 and Supplementary Table S3). However, if a male did not start the wing vibration within 10 minutes, we did not include it in the rest of the analysis, as previously reported^21,22^. Our results revealed that the courtship latency did not show a significant difference among males with the 0°, Right 45°, and 180° angle deviation and control males (Kruskal-Wallis test, *P* = 0.3559) (Fig. 5c, Supplementary Table S7, and Supplementary Table S8).

The “courtship index” (CI) is generally used to quantify how enthusiastically a single male courts a single female for the first 10 minutes^21,22,24^. The CI is calculated as the percentage of time spent in all of the components of courtship, including tapping, following, wing extension and vibration, licking, and attempted copulation^19,20,22,24,25^. Using the same video data, we obtained the CI and compared the values among males with the 0°, Right 45°, and 180° angle deviation and control males (backcrossed *w*^1118^). We did not find a significant difference in the CI among the males with genitalia with angle deviation (Fig. 5d, Supplementary Table S7 and Supplementary Table S9). However, the CI of control males was lower than that of males with genitalia classified as 0°, Right 45°, and 180° angle deviation, which carried the *Myo31DF*^*K2*^ mutation (Fig. 5d, Supplementary Table S7 and Supplementary Table S9). Although we do not know the reason for the difference in CI at this point, it is possible that *Myo31DF* has a weak phenotype in courtship behavior. Alternatively, our backcross may not have been sufficient to uniformalize the genetic background. Nevertheless, our results demonstrated that the deviation in genital rotation did not affect the courtship latency or CI. Therefore, we concluded that the decrease in successful copulation was the main cause of the reduction in fecundity associated with angle deviation of the male genitalia.

In addition to the courtship latency and CI, we examined whether the male-above mating position was influenced by angle deviation of the male genitalia. We examined all of the video data of the courtship assay involving the males with genitalia classified as Right 45° and 180° angle deviation. However, we did not observe any atypical mating positions attempted by these males, in any case examined (see Supplementary videos 2 and 3 as typical examples). Therefore, regardless of the dorsoventral direction of the male genitalia, the mating behaviors leading to the male-above position were fixed. Taking all of our results together, we demonstrated that the normal dorsoventral direction of the male genitalia is a prerequisite for successful mating in the male-above mating position in *D. melanogaster*. Therefore, our findings suggest that cooperative evolution can exist between mating behavior and male genitalia rotation in this species.

## Discussion

Cooperative evolution between organ morphology and behavior is a well-established concept^1–6^. Phylogenetic analyses in various animal groups have demonstrated remarkable coincidences between organ morphology and behavior^1–6^. In this study, we sought to obtain evidence supporting the idea that mating behaviors cooperatively evolved with the rotation of male genitalia in dipteran insects.

In these insects, the evolution from the end-to-end to the male-above position should result in an incompatibility in the dorsoventral direction of the male genitalia with respect to that of the female genitalia^4,5^. It was previously proposed that this incompatibility was overcome by the additional 180° rotation of the male genitalia (in total, a 360° rotation called “circumversion”)^4,5,7^. In this study, we showed that males with genitalia resulting from complete circumversion had a great advantage for reproduction over those with deviation in the rotation angle of the genitalia. Our results indicated that the proper dorsoventral direction of the male genitalia is a prerequisite for successful copulation in the male-above mating position in *Drosophila*. Therefore, our results are consistent with the previously proposed idea that the mating position of dipterans evolved cooperatively with changes in the rotation angle of the male genitalia^4,5^.

In this analysis, we found that males with genitalia classified as the Left 45° and Right 45° angle deviation still copulated and had offspring (Fig. 3c,d). Therefore, males may be able to overcome a small aberration in the dorsoventral direction of their genitalia, although we could not exclude the possibility that females also contribute to this adjustment. In *D. melanogaster*, the males have genitalia sensilla, including bristles on the genital claspers, which detect the position of the mating partner and contribute to the male’s proprioception^26^. Acebes *et al*. showed that ablating the genital sensilla caused a reduction in mating frequency and decreased the male’s ability to detect his position on the female’s back^26^. Therefore, in our experiments, it is possible that the sensitivity of the male genitalia contributed to the adjustment to a small aberration in the dorsoventral direction of the male genitalia. However, Acebes *et al*. also showed that ablating the genital sensilla does not affect the male’s attempt to mount the female back, demonstrating that the male’s attempt to copulate on the female’s back is an unalterable component of male-above mating^26^. This idea is consistent with our observation that the males with genitalia classified as Right 45° and 180° (upside down genitalia) angle deviation still used the male-above mating position, and no other mating positions, in all of the video data examined (n = 14 and n = 10, respectively) (see Supplementary videos 2 and 3 as typical examples). Upon an alteration in body structure, mating behaviors are also found to be inflexible in other insects. For example, the South American four spot-roach, *Eublaberus distanti* has a highly ritualized mating behavior, which requires male wing rise^27^. Males in which the wings have been partially or completely surgically removed still perform the full sequence of mating behaviors and complete their courtship ritual^27^. Therefore, in cases in which a particular body structure and mating behavior are cooperatively required to achieve successful mating, the mating behavior is often inflexible to adjust to a change in body structure, as found here for the dorsoventral direction of the male genitalia.

In this study, we obtained *Myo31DF* mutant male flies with genitalia exhibiting various deviations in the rotation angle. In our classification of the angle deviation, the Right 45° and Left 45° males had a deviation of the same angle, which was left-right inversed. However, there is a 270° difference in the amount of rotation needed to achieve these deviations; the Right 45° and Left 45° angle deviations were achieved by 315° and 45° counterclockwise rotations, respectively. However, we did not find a noticeable difference between the success rates of reproduction between these two categories (Fig. 3c,d, Supplementary Table S2). These results suggested that the amount of genital rotation itself does not impact reproduction; rather, the final dorsoventral angle of the genitalia resulting from the rotation is critical for successful copulation. In this context, Huber *et al*. proposed based on phylogenetic analyses that morphological asymmetry itself is not advantageous for reproduction in spiders and insects, but rather the newly adopted mating position is^4^. Their idea is consistent with our experimental observation that the degree of genitalia rotation did not affect reproduction if the final dorsoventral direction of the male genitalia was the same.

In our experiments, males with genitalia demonstrating all classes of angle deviation were obtained from *Myo31DF*. We found that the males with genitalia classified as 0°, Right 45°, and 180° angle deviation and control males, whose genetic background was unified with *Myo31DF* through five backcrosses, did not show significant differences in courtship latency or in the percentage of males initiating the courtship (Fig. 5c and Supplementary Table S8). However, although the CI was not significantly different among males with genitalia classified as 0°, Right 45°, and 180° angle deviation, the CI of the *w*^1118^*/Y* control males was lower than those of the males with angle deviations in their genitalia (Fig. 5d, Supplementary Table S7 and Supplementary Table S9). Therefore, the *Myo31DF*^*K2*^ mutation may affect the CI. In support of this possibility, *Myo31DF* was recently shown to be involved in the formation of neuromuscular junctions and in TNF signaling^17,18^. Alternatively, the genetic background of the control males may not have become completely unified with that of *Myo31DF*. However, all of our conclusions were based on comparisons between males with genitalia showing the classified angle deviations as well as the normal direction, all of which were on the same*Myo31DF* genetic background. Therefore, the observed difference in the CI of the control males does not affect our conclusion that the normal dorsoventral direction of the male genitalia was highly preferable for successful copulation.

Although our hypothesis is based on the single example of *D. malanogaster*, it supports the idea that the male–above position evolved cooperatively with the additional 180° rotation of the male genitalia from the ancestral end-to-end position, which was proposed before^4–7^. However, we also found that *Drosophila* males have a very limited ability to adjust to a deviation in the direction of their own genitalia. In contrast, the ancestral species that used the end-to-end position might have had high flexibility in the mating position, which gave it the ability to tolerate the future additional 180° rotation of the male genitalia. That is, such flexibility might have subsequently allowed cooperative evolution between the mating position and the rotation of male genitalia. Alternatively, the evolution from the end-to-end to male-above position might have gone through an intermediate step, such as the false-male-above or male-above with flexed male abdomen position^4–6,28^. In this scenario, the mating position evolved before the additional 180° rotation of the male genitalia. To evaluate these ideas, further phylogenetic analyses of the relationship between mating behavior and male genitalia rotation are required.

## Materials and Methods

### Fly stocks

Flies were cultured on standard medium at 25°. Canton-S was used as the wild-type strain. *Myo31DF*^*K2*^ is a null mutant of *Myo31DF*^12^. In all experiments, the *Myo31DF*^*K2*^ mutant line carrying *w*^1118^ was used (*w*^1118^; *Myo31DF*^*K2*^). *white*^1118^ (*w*^1118^) is a previously described loss-of-function allele of *w*^29,30^.

### Collection and classification of *Myo31DF* homozygous males with deviations in the rotation angle of their genitalia

In the majority of *Myo31DF*^*K2*^ homozygous males, the genitalia rotate 360° counterclockwise, which is the left-right inversed direction of their wild-type counterpart^12,14^. However, the rest of the males had genitalia whose rotation stopped before it was completed. The deviation in the rotation angle of the male genitalia (angle deviation) was defined as previously described with slight modifications^14^. The angle formed by the midline of the abdomen and the dorsoventral line that passes though the anus and penis (middle of the claspers) was measured with Image J (http://rsb.info.nih.gov/ij/) on a photograph of the genitalia of each male (VB-7010, Keyence) (Fig. 2a,b). Based on this angle, the angle deviations were classified into eight groups: 0° (Left 22°-Right 22°), Right 45° (Right 22°-Right 67°), Right 90° (Right 67°-Right 112°), Right 135° (Right 112°-Right 157°), 180° (Right 157°- Left 157°), Left 135° (Left 157°-Left 112°), Left 90° (Left 112°-Left 67°), and Left 45° (Left 67°-Left 22°) (Fig. 2c).

### Evaluating the effect of male genitalia angle deviation on fecundity

Wild-type and *Myo31DF*^*K2*^ homozygous females were collected six to eight hours after eclosion and kept in fresh medium for 3–5 days. To confirm that all females were virgin, the medium in which these females were kept was incubated for at least another week at 25 °C to assure that they did not bear progeny.

One male classified according to its genitalia angle deviation and three virgin females were then cultured together in a vial of standard medium for four days. Under these conditions, the wild-type and *Myo31DF*^*K2*^ homozygous males with genitalia in the normal direction successfully mated with wild-type virgin females and yielded progenies at 100% (Fig. 3a,b). After four days, the males were recovered from the culture, their genitalia were photographed, and the angle deviation was measured as described above. To collect the potential progenies, the females were kept in the same medium for another 5 days and then discarded, followed by further incubation of the medium for another 5 days. If progeny were found in the medium, the mating was regarded as successful.

### Courtship assays

To analyze the potential influences of the male genitalia angle deviation on mating behaviors, we measured a set of courtship parameters: copulation success rate, courtship latency^21–23^, courtship index (CI)^19,20,22,24,25^, and reproduction success rate (fecundity). All females were wild type. The males were *w*^1118^*/Y*; *Myo31DF*^*K2*^ with genitalia with classified angle deviations and the control *w*^1118^ line that was backcrossed with *w*^1118^*/Y*; *Myo31DF*^*K2*^ (see backcross method below). The flies used in these experiments were collected six to eight hours after eclosion as virgins using carbon dioxide anesthesia, and males were collected and roughly classified according to angle deviation (following Fig. 2a–c). Until used for the tests, the female and male flies were maintained individually for another three to five days and five to seven days, respectively, in vials containing standard medium, under a 12-hour light-dark cycle^19,31^.

For courtship assays, a wild-type virgin female was placed in a small chamber (8-mm in diameter and 3-mm high) followed by the addition of a male with genitalia in the normal direction or classified according to angle deviation, and their courtship behavior was video-recorded for one hour or extended to the end of copulation if they were copulating at the end of the one-hour period, using an HC-V550M video camera (Panasonic). The copulation success rate was the percentage of males that mounted a female continuously for more than one minute during the one-hour courtship assay. To facilitate this analysis, we wrote image analysis software, in which the outlines of flies were obtained by the binarization of video images, and these images were arranged in chronological order (see video analysis program below). Using this analysis, we could efficiently detect when flies were copulating, although it was not sensitive enough to define each step of the courtship. This image analysis software also enabled us to observe the video image that corresponded to every time point of the processed data, which allowed us to confirm successful copulation from snapshots of the video data. After taking the video, the males were recovered, their genitalia were photographed, and the angle deviation was measured (VB-7010, Keyence).

Courtship initiation was defined as the beginning of male wing vibration^23^. Courtship latency was defined as the period of time from the observation start time to the beginning of the first wing vibration (courtship initiation)^23^. Males that did not start the wing vibration were excluded from this analysis, although there were very few of them (Supplementary Fig. S1). The courtship index (CI) was calculated as the percentage of time that was spent on the courtship, including orientation, tapping, following, wing extension and vibration, licking, and attempted copulation for 10 minutes, as described previously^19,20,22,24,25^. We used a stopwatch to record the total time in which a male engaged in courtship activity from video-recordings obtained as described above^21,25^. Males that did not copulate for 10 minutes were excluded from this analysis, as described previously^22^. The percentage of males that initiated courtship is shown in Supplementary Fig. S1. The reproduction success rate represents the percentage of males that yielded offspring after the one-hour courtship analysis. After the courtship analysis, each female was recovered, separately maintained in standard medium for five days, and discarded. The vials were incubated for another five days to determine whether offspring were present.

### Backcross to generate the control *w*^1118^ line

As a control for the courtship assays, we used *w*^1118^ hemizygous males, because the *Myo31DF*^*K2*^ line also carries *w*^1118 12^. To make the genetic background as uniform as possible, we backcrossed *w*^1118^ with *w*^1118^*/Y*; *Myo31DF*^*K2*^ five times. To distinguish between wild-type *Myo31DF* and the *Myo31DF*^*K2*^ mutation, we conducted genomic PCR. At every generation, a single pair in which the female could be homozygous or heterozygous for *Myo31DF*^*K2*^ was recovered. Genomic DNA was extracted and used as a template for PCR to check the genotype using two sets of primers.

The wild-type *Myo31DF* gene was amplified by a set of forward and reverse primers (5′- TGCAATGCCTTCAAGACCC -3′ and 5′- GCCGTAAATATCCAGCACTCC-3′) that yielded a 472-bp product. The PCR reaction was conducted according to the manufacturer’s instructions (Ex Taq, Takara, Japan), with modifications of the cycle number from 30 to 34 and annealing temperature from 55 °C to 52 °C.

The genotype of the final *w*^1118^ line, which carried the wild-type *Myo31DF* gene, was confirmed by the same PCR reaction and subsequent analysis. The wild-type *Myo31DF* locus was amplified by a set of forward and reverse primers (5′-TGGGAACACGTAATGCGTAA-3′ and 5′-TCTGCACTCGATTTGTCAGG-3′) that yielded a 484-bp product. To exclude the line with the *Myo31DF*^*K2*^ mutation, this gene was amplified by a set of forward and reverse primers (5′-TAATCCGCCGCAAACGAAAC-3′ and 5′-TTCGTTCTTTTCGTCGGCCA-3′) that yielded a 431-bp product. In both experiments, the PCR reactions were conducted according to the manufacturer’s instructions (Ex Taq, Takara, Japan), with modification of the cycle number from 30 to 34. In PCR reactions for the wild-type *Myo31DF* and *Myo31DF*^*K2*^ alleles, the annealing temperature was 50 and 52 °C, respectively. A *w*^1118^ line that carried only the wild-type *Myo31DF* and not the *Myo31DF*^*K2*^ mutation was established and used as a control in the courtship assays.

### Video analysis program

To facilitate the courtship assays, we generated a program for processing the images from the video data. A time code was inserted into each video image (Fig. 4b), and then images were collected every 10 seconds. The collected images were binarized, and the shapes of the flies were automatically selected (Fig. 4c). The selected shapes were stacked and arranged in 3D space along a time line (Fig. 4d). To identify time points on the 3D image, a moving bar was inserted, which was synchronized with the 2D images (yellow lines in Fig. 4d). Using the slide bar indicated by these yellow lines on Maya version 2018 (Autodesk, San Rafael, CA), the 2D (video image) and 3D constructed images could be correspondingly identified at any time point (Fig. 4d and Supplementary Fig. S2). With this system, the start and end points of copulation could be quickly identified. After identifying these time points from the 3D constructed images, we confirmed them using the video images.

### Statistical analysis

Statistical analyses and graph constructions were performed in R 3.3.1 (R Foundation for Statistical Computing, Vienna, Austria). To evaluate the statistical significance of the differences in reproduction success rates, Fisher’s exact test was performed. In the courtship latency analysis, a nonparametric Kruskal-Wallis test was performed, and the results were evaluated by a Steel-Dwass test to judge the significance of the difference between each group. The CI was analyzed with One-Way Analysis of Variance (ANOVA) and evaluated by Tukey test to judge the significance of the difference between each group. A *P*-value < 0.05 was considered to indicate a significant difference.

## Electronic supplementary material

Supplementary materials

Supplementary movie 1

Supplementary movie 2

Supplementary movie 3
