# Supplementary material for: Proper direction of male genitalia is prerequisite for copulation in Drosophila, implying cooperative evolution between genitalia rotation and mating behavior
Source: Sci Rep. 2019 Jan 18;9:210. doi: 10.1038/s41598-018-36301-7 (PMC6338758; doi:10.1038/s41598-018-36301-7)
Supplement: Supplementary file 1 — Supplementary materials [file 41598_2018_36301_MOESM1_ESM.pdf]

Proper direction of male genitalia is prerequisite for copulation in *Drosophila*, implying cooperative evolution between genitalia rotation and mating behavior

Momoko Inatomi[1], Dongsun Shin[1], Yi-Ting Lai[1], and Kenji Matsuno[1*]

[1]Osaka University, Graduate school of Science, Department of Biological Sciences, Osaka, 560-0032, Japan.

Supplementary Tables

Table S1. Reproduction rates of males with genitalia in the normal dorsoventral direction after various days of mating

	Reproduction rate			
	Cross period (days)			
Male line	1	2	3	4
Wild type	100% (n=11)	91.7% (n=12)	83.3% (n=12)	100% (n=11)
Myo31DF^{K2}	90.9% (n=11)	100% (n=11)	100% (n=11)	100% (n=10)

Females were wild type. The number of males analyzed is shown in parenthesis.

Table S2. Reproduction success rate of males with genitalia exhibiting the eight classes of rotation deviation

	Rate of reproduction							
	Deviation class of male genitalia							
Female	0°	Right 45°	Right 90°	Right 135°	180°	Left 135°	Left 90°	Left 45°
Wild type	66.2% (n=68)	33.9% (n=59)	1.96% (n=51)	3.18% (n=63)	0% (n=52)	0% (n=43)	3.03% (n=33)	46.2% (n=26)
Myo31DF^{K2}	75.0% (n=56)	60.0% (n=55)	9.80% (n=51)	0% (n=54)	0% (n=45)	0% (n=25)	0% (n=10)	65.0% (n=20)

The number of males analyzed is shown in parenthesis.

Table S3. Percentage of males with genitalia with angle deviations that initiated wing vibration in the single pair courtship assay

Angle deviation	Number analyzed	Males initiating the wing vibration
0°	18	100%
Right 45°	16	87.5%
180°	10	100%
Control	17	94.1%

Table S4. Copulation and reproduction success rates of males with genitalia exhibiting different angle deviations in the single pair courtship assay

Angle deviation	Number analyzed	Copulation success rate	Reproduction success rate
0°	18	88.9%	83.3%
Right 45°	14	7.14%	7.14%
180°	10	0%	0%
Control	16	56.3%	56.3%

Females were wild type.

Table S5. Fecundity of males with genitalia with angle deviations that successfully copulated in the single pair courtship assay

Angle deviation	Number analyzed	Rate of successful copulation
0°	16	93.8%
Right 45°	1	100%
180°	N.A.	N.A.
Control	9	100%

Females were wild type.

Table S6. Fecundity of males with genitalia with angle deviations that failed to copulate in the single pair courtship assay

Angle deviation	Number analyzed	Rate of successful copulation
0°	2	0%
Right 45°	13	0%
180°	10	0%
Control	7	0%

Females were wild type.

Table S7. Courtship latency and courtship index of males with genitalia exhibiting different angle deviations

Angle deviation	Number analyzed	Mean ± S.E.	
		Courtship latency (seconds)	CI
0°	18	114.2±11.3	0.5616±0.0340
Right 45°	14	95.29±19.4	0.5823±0.0391
180°	10	108.7±30.03	0.4798±0.0570
Control	16	148.2±24.3	0.2708±0.0449

Females were wild type.

P-value for courtship latency was calculated with the Kruskal-Wallis test (P=0.3559).

P-value for CI was calculated with ANOVA (P=0.000002852).

Table S8. Steel-Dwass test of the mean courtship latencies

Pair compared	p-value
0° angle deviation-Right 45° angle deviation	0.4030
0° angle deviation-180° angle deviation	0.7168
0° angle deviation-Control	0.8727
Right 45° angle deviation-180° angle deviation	0.9998
Right 45° angle deviation-Control	0.6231
180° deviation-Control	0.7330

Table S9. Tukey HSD test of the mean CI values

Pair compared	p-value
0° angle deviation-Right 45° angle deviation	0.9790
180° angle deviation-0° angle deviation	0.5775
Control-0° angle deviation	0.0000161
Right 45° angle deviation-180° angle deviation	0.4090
Control-Right 45° angle deviation	0.0000128
Control-180° angle deviation	0.01169

Supplementary video 1.

Typical video data of the courtship assay involving a wild-type virgin female and a male that had genitalia with 0° angle deviation. The flies in this video copulated successfully.

Supplementary video 2.

Typical video data of the courtship assay involving a wild-type virgin female and a male that had genitalia with Right 45° angle deviation. The flies in this video copulated successfully.

Supplementary video 3.

Typical video data of the courtship assay involving a wild-type virgin female and male with genitalia with 180° angle deviation. The flies in this video failed to copulate.

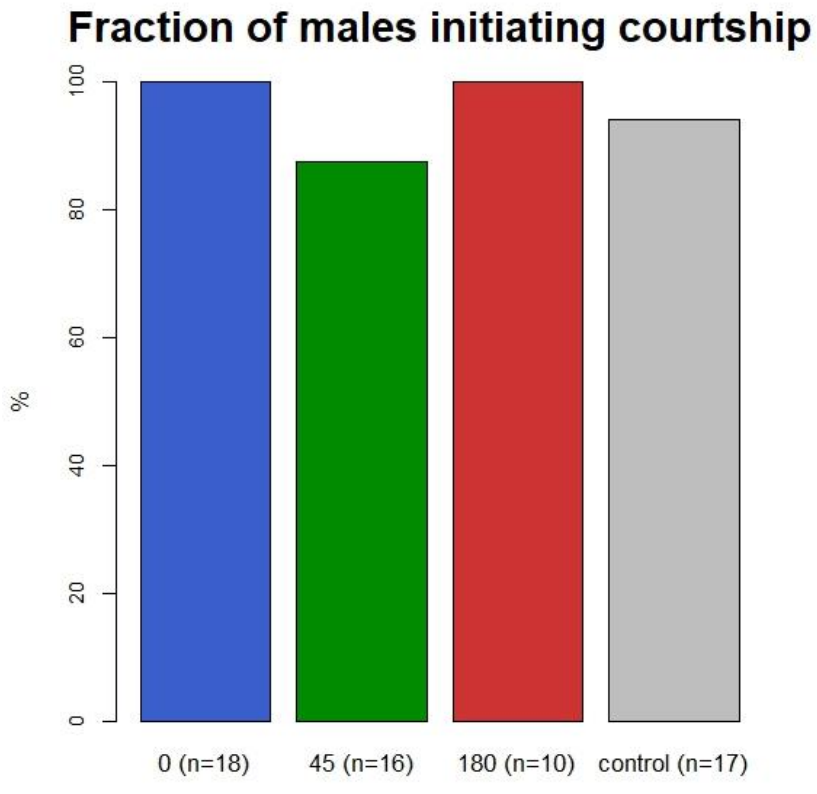

Supplementary Figure S1.
Percentage of males that initiated wing vibration during the first 10 minutes in the mating behavior assay. A wild-type virgin female was mated with a male that had genitalia with angle deviation classified as 0° (0), Right 45° (45), or 180° (180) or with a control male (control). The number of males examined is shown in parentheses as n.

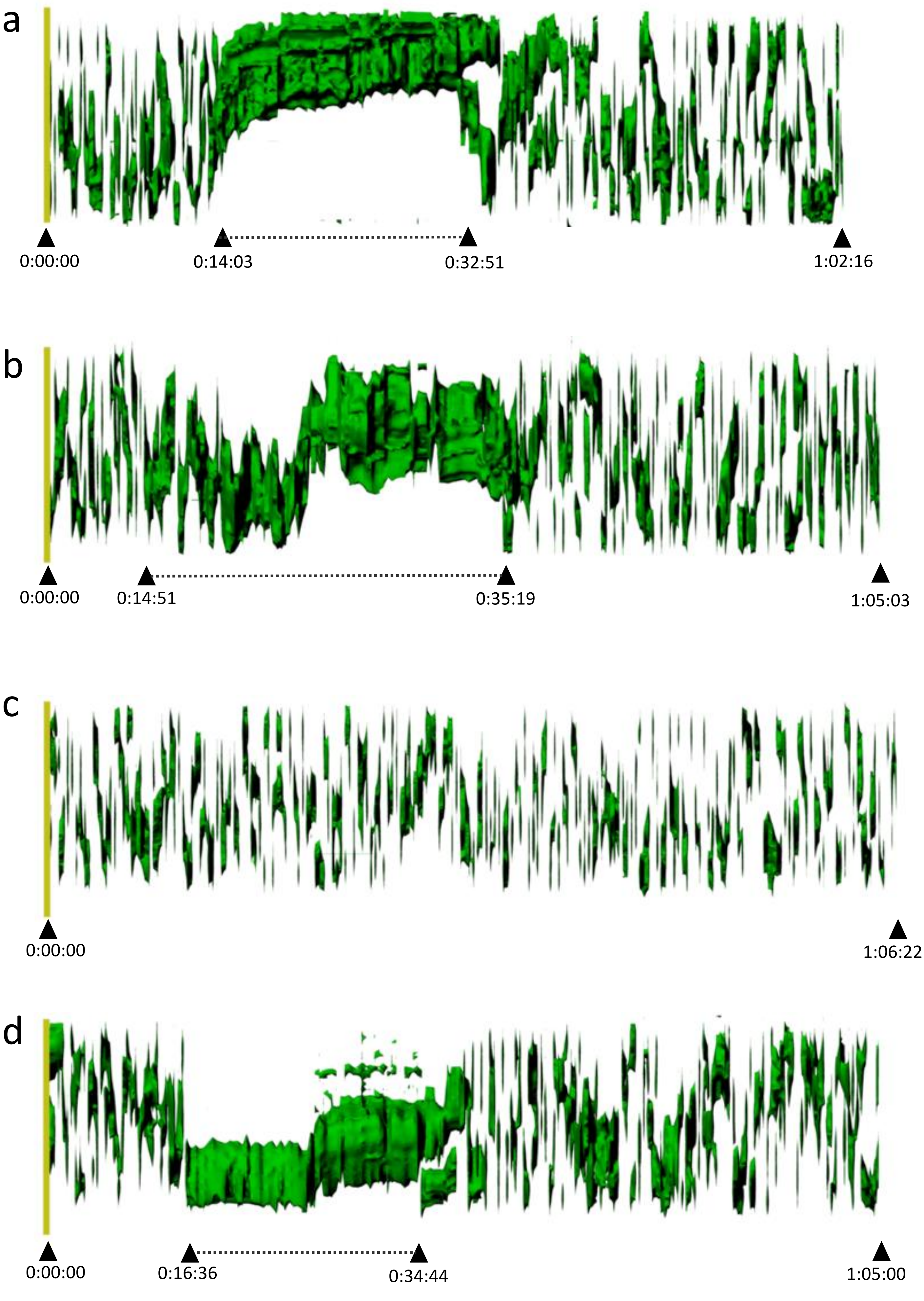
a
0:00:00
0:14:03
0:32:51
1:02:16
b
0:00:00
0:14:51
0:35:19
1:05:03
c
0:00:00
1:06:22
d
0:00:00
0:16:36
0:34:44
1:05:00

Supplementary Figure S2.
Typical results of mating behavior analyzed by the novel image-processing program. A wild-type virgin female was mated with a male that had genitalia with angle deviation classified as 0° (a), Right 45° (b), or 180° (c) or with a control male (d). The left side of the images corresponds to the start of the courtship assay. Black triangles show time points. In a, b, and d, the pairs showed successful copulation, because the images of the female and male overlapped each other (broken lines between arrowheads) for more than 1 minute.
